# Is It Essential for Occupational Health and Safety Experts to Know the English Language? Results From Several Studies in Latvia

**DOI:** 10.3389/fpubh.2022.833620

**Published:** 2022-03-15

**Authors:** Linda Matisāne, Linda Paegle, Lāsma Akūlova, Maija Eglı̄te, Ivars Vanadziṇš

**Affiliations:** Institute of Occupational Safety and Environmental Health, Riga Stradiņš University, Riga, Latvia

**Keywords:** occupational health and safety expert, language skills, occupational health and safety competence, COVID-19, occupational health service

## Abstract

**Background:**

Poor knowledge of the language has been identified as a barrier to up-to-date occupational safety and health information, however, this question has not been addressed in the context of occupational safety and health expert competence in providing advice for employers in a small, non-English speaking country.

**Objectives:**

To analyze the available data on the use of languages for searching professional information by occupational safety and health (OSH) experts, and the sources of information on COVID-19 during the first wave of the pandemic in Latvia, and to assess if the knowledge of the English language among these experts is sufficient to react rapidly and effectively in case of emergency.

**Methods:**

Data were compiled from several different sources: three web-based surveys of occupational safety and health experts (data from 2006, 2010, and 2018) and ten focus group discussions with employers and occupational safety and health experts (data from 2020).

**Results:**

The results of the surveys show that between 2006 and 2018, the percentage of respondents using only one language (Latvian) for searching professional information in occupational safety and health has increased from 25 to 35.3%. In 2018, the English language was mentioned by only 42.8% of respondents and Russian by 46.8%. During the focus group discussions, the use of English was mentioned for obtaining trustful information from international organizations, for analysis of information received within international companies, for searching international experience, and for finding county-specific information.

**Discussion:**

Our study shows that knowledge of the English language for occupational safety and health experts working in Latvia is not sufficient. The companies providing external occupational safety and health services should establish a well-functioning internal training system to provide their non-English speaking experts with up-to-date information. Occupational safety and health-related non-governmental institutions should strengthen their capacity in sharing information related to different occupational safety and health aspects into the national language (Latvian in this particular case).

## Introduction

Before the COVID-19 pandemic, it has already been recognized that the changes in the work environment have required today's OSH experts and their workplace functions to change (1). Therefore, these experts have to broaden their range of skills, knowledge, and behavior that are essential to influence and to drive changes in the work environment (2).

To maintain the health of individuals, families, and communities, everyone relies on the health information available to them (3). This means that to provide the best advice on the prevention of workplace hazards, OSH the experts need the best available knowledge on the factors influencing the health and safety of workers. Language, as a barrier to access the up-to-date OSH information, has already been addressed in the OSH context for many years. Insufficient language knowledge has been identified as a reason for a lower understanding of the basic OSH requirements and procedures, thus, resulting in poor work practices among immigrant workers and ethnic minorities (4, 5). This is mainly related to the fact that most of the employers offer OSH training in the languages spoken by a major part of the population within the country, therefore, the information is available, but might not be understandable to the migrant workers.

If we adopt this statement to the situation at the beginning of the COVID-19 pandemic in Latvia, we obtain the following hypothesis: a lower COVID-19 literacy in OSH experts was caused by the lack of information in the Latvian language, although this information was available in other languages (e.g., English). It is clear that the information on the SARS-CoV-2 virus and the methods to mitigate the spread of this virus were missing in the world, but it is also clear that more information from international organizations (like the World Health Organization, the International Labour Organization, and the European Agency for Safety and Health at Work) and good practice examples from companies were available in English than in Latvian, which is the official language of the country with ~1.87 million inhabitants (6).

Therefore, we decided to analyze the available data to assess the use of languages for searching professional information by OSH experts and on COVID-19 during the first wave of the pandemic in Latvia. Another aim of our study was to check our hypothesis; we assumed that the COVID-19 pandemic has shown the need for knowledge of the English language of OSH experts to react rapidly and effectively to the new and emerging risk factors.

## Materials and Methods

No special data to draft this article were gathered; data for this purpose were compiled from four different sources gathered with quantitative and qualitative research methods: data from three web-based questionnaires for OSH experts in Latvia (in 2006, 2010, and 2018) and transcripts from focus group discussions of study on working life with COVID-19 (carried out in 2020) were used to analyze the opinion of employers and their representatives, who are the OSH experts. Ethical approval for the study was granted by the Ethics Commission of Riga Stradiņš University (protocol No. 6-1/08/16, 23 July 2020) before the recruitment of the focus group participants.

### Surveys of OSH Experts

#### Study Design and Recruitment of Participants

Four consecutive national surveys of *Work conditions and risks in Latvia* had been conducted in 2006 (7), 2010 (8), 2013 (9), and 2018 (10). However, the opinion of OSH experts (persons with a post-graduate degree in OSH) has been surveyed only in 2006, 2010, and 2018. These surveys aimed to gather evidence on the prevalence of workplace hazards, occupational diseases, and accidents at work that would serve as a basis for effective decision-making in the creation and adjustment of employment and social policy programs.

The same survey methodology used for years was utilized; the web-survey answers were gathered from OSH experts on different OSH-related aspects. It applied a non-probability sampling method. Survey participants were recruited using a snowball sampling method. Public announcements, social media advertisements, direct emails, employers' non-governmental organizations, personal contact networks, and higher educational establishments providing postgraduate education in OSH were used to share the web link of the questionnaire in Latvian. Every single person with access to the internet was able to fill in the questionnaire, but, at the beginning of the web survey, a filtering question was applied to recruit only persons who have obtained a degree in OSH. In 2006, 86 persons responded to the questions, the number of respondents in 2010 and 2018 are 211 and 202, respectively. More information on these surveys is given in Table 1.

**Table 1:** Description of the *Work conditions and risks in Latvia* study samples.

**Survey**	**Total number of the study population**	**Survey periods**	**The tool used to gather answers**
2006	86	19.04.2006–15.07.2006	Webropol
2010	211	09.02.2010–05.03.2010	Webropol
2018	202	04.06.2018–22.08.2018	n.a.*

* *n.a., information not available*.

At the beginning of all the three web-survey, written information on the purpose of the study was provided, therefore, participants agreed to participate in the survey by voluntarily proceeding to further questions.

#### Study Variables

For this article, only one main question from the surveys was used: “In which languages do you search information on OSH?”. The respondents could select several answers from the following options: “In Latvian,” “In English,” “In Russian,” “In German,” and “Other.” If the person did not answer this question, his/her answers were excluded from further analysis (in 2006–2 persons, in 2010–1 person, and 2018–1). For a more specific analysis, several other responses were excluded, mainly because the person mentioned the use of other languages or did not specify which other languages were used (in 2006–7 persons mentioned German, 1—Swedish, 1—Spanish; in 2010–8 persons mentioned German, 1—other, not specified; in 2018–2 persons mentioned German). The German language was excluded from further analysis due to the low number of respondents mentioning this language in the newest survey in 2018.

The work experience in OSH and the workplace of respondents were used as independent variables. The work experience was measured by asking respondents “How long do you work in OSH?”. The respondents could choose from the following answers: “1 year,” “2–5 years,” “6–10 years,” “11–15 years,” “more than 15 years,” and “at this moment I do not work in OSH” (for analyses, the answers “1 year” and “2–5 years” were combined in a group “1–5 years”; the answers “11–15 years” and “more than 15 years were combined in a group of “more than 11 years”). The workplace of the respondents was identified by asking the question: “Where do you work?”. The following answers were offered: “In a company providing external OSH services,” “In the OSH department of one company (there are more than one OSH expert in the company),” “In one company as an internal OSH expert (the only OSH expert in this company),” “In several companies as an internal OSH expert,” “In several companies as an external OSH expert (service provider),” “In-state authority (Ministry of Welfare, State Labour Inspectorate),” and “Other.” For all the questions, an answer “I don't know/I don't want to answer” was possible, therefore, these answers were excluded from the analysis of the relevant variable. A filter was applied for respondents who have reported that they did not work in OSH at the moment of the survey, therefore, they did answer the question on the current workplace.

#### Data Analysis

Frequency analyses (percentages, distribution) were used to describe the data. The average age of these respondents and gender distribution is not available as such information was not gathered during the original studies. The analysis was conducted using the IBM SPSS Statistics 27 (IBM Corporation, Armonk, New York, NY, USA) software.

### Focus Group Discussions With Employers and OSH Experts

#### Study Design and Recruitment of Participants

For this research, the gathered qualitative data during 10 focus group discussions on working life during the COVID-19 pandemic were used. All the focus groups were held during September and October of 2020, just before the 2nd wave of the COVID-19 pandemic in Latvia. The focus group participants were either employers, their representatives, or OSH experts with a postgraduate degree in OSH. For recruitment of the voluntary focus group participants, public announcements, social media (Facebook and Twitter) posts, local employers' non-governmental organizations, personal contact networks, and national labor inspectorate were used. Before the discussions, the purpose of the study was explained to the participants, and verbal consent was obtained from them. No monetary compensation was provided to the participants.

#### Focus Group Discussions of Employers

Eight of the focus group discussions were organized to gather information from employers. In total, 65 employers from companies of different sizes and regions participated in the discussion (the smallest group had 5 participants, and the biggest had 11 participants). Based on the economic structure of Latvian companies, the representatives of the companies were categorized into 2 groups for this study. The representatives from organizations with <100 workers were classified in the group of small and medium-sized companies, but representatives from the organizations with 100 or more workers were classified as large. While recruiting the focus group participants, the affiliation of the possible participant was asked to ensure that he/she has the affiliation allowing him/her to express the opinion of the employer. If the person did not comply with this requirement, he/she was not included in the focus group. If the applying person did not match the criteria for the specific group (e.g., geographical location of the company or the company size), he/she was invited to take part in the relevant group or excluded from participation in the focus group discussions.

#### Focus Group Discussions of OSH Experts

Two different focus group discussions for OSH experts were organized: 12 external OSH service providers were included in one group, and 12 companies with internal OSH experts in another. While recruiting, the status of the applying OSH expert was checked through their affiliation (in case of company internal OSH experts) or the official online list published on the website of the Ministry of Welfare (in case of registered external OSH service providers). If the applying person did not match the requirements for the specific group (e.g., internal/external OSH expert), he/she was offered to participate in the other group or excluded from participation in the focus group discussions.

#### Procedure of the Focus Group Discussions

A standardized procedure was used for all focus group discussions. Because of the epidemiologic restrictions implemented by the Latvian government to mitigate the COVID-19 pandemic, a mixed interviewing method was used; some of the participants were on-site, others used online meeting platforms, such as Zoom or MS Teams. The experienced and trained moderators led the focus group discussions (interviewers—IV, IA, and SR). They were facilitated by a note-taker to make the transcribing process easier. The focus group discussions strictly followed the structured research protocol guidelines with logically proceeding groups of questions. Research protocol guidelines were pre-tested with persons familiar with OSH topics, but were not involved in the study neither as researchers nor focus group participants.

The topic related to searching information on COVID-19 was included toward the end of the discussions when the general topics applying to all workers were already covered. According to the structured guidelines, participants were initially asked, “Where did you personally search professional information on measures to be implemented in the workplace for mitigation of spreading on COVID-19 virus? Did you use the website of the State Labour Inspectorate, the Ministry of Welfare, the Ministry of Economy, [national working life portal] www.stradavesels.lv, client service phone of the State Labour Inspectorate, information published social media accounts of the State Labour Inspectorate other sources?... Did you search for information in English or any other language? Recommendations of which organizations did you use?”. As part of the discussions held online, the moderator used a PowerPoint presentation with written questions which were shown on the screen when the relevant questions were discussed.

After receiving permission from all participants, the focus group discussions were recorded. The recording aimed to facilitate the transcribing process and to ensure that the information is matched correctly. The recordings and transcripts are safely stored according to the data protection rules of Riga Stradiņš University. The group discussions lasted between 115 and 152 min each, the topics related to searching for professional information were covered in ~12 min per focus group.

#### Procedure of the Focus Group Discussions

After preparing the anonymized transcripts, the participants were de-identified manually by an independent researcher (LA) who did not participate in discussions and content analysis of the results. A careful and systematic analysis, including coding, and interpretative work, was done by two independent researchers for obtaining results at the group level. Both researchers providing the coding are OSH experts with more than 20 years of experience and with a background in occupational medicine (LM and IV) who were advised by another researcher holding a master's degree in public health (LP).

Initially, both experienced OSH researchers (LM and IV) together read through the data and suggested tentative categories and subcategories, which were based directly on the data itself without building them on theoretical considerations. Such a decision was made as it seemed that this approach better fitted the research question. Then, the same researchers separately coded the transcript of the discussion of the external OSH experts. After that, both researchers compared their analysis and agreed on the categories and subcategories, which were used for further coding of other transcripts. The buildup of subcategories was also continued while the analytical process of all 10 focus group discussion transcripts. Then, the subcategories were refined by collapsing and merging the initial ones into the final version. Finally, the third independent researcher (LP) reviewed all transcripts to verify the findings and to visualize them to be presented as results.

The supporting quotes were selected in all stages of the coding and reviewing process. Then, all three experts discussed and agreed on the best fitting ones, which were later included in the section of the results as examples to describe the different ways the responses were given. For the selected quotes from the employers' focus group discussions, the size of the quoted company and region is given (Riga, suburbs of Riga represent the biggest city of the country and its surroundings; all others are regions of Latvia); for OSH experts, it is mentioned if the quoted person works as an internal OSH expert or OSH service provider (referred as “internal” or “external,” respectively).

In addition to identifying categories and subcategories, the researchers repeated the analyses of the transcripts to find places and supporting quotes in the transcripts, where the use of languages other than Latvian for obtaining information during the COVID-19 pandemic was mentioned. Based on the formulation of the question used during the focus group discussions (“Did you search information in English or any other language?”) and the results of the content analyses, the experienced researchers agreed that indirect mentions of the use of English will be captured along with direct mentions. The indirect mentions were used for cases when it was clear that the relevant information from the mentioned sources could not be obtained in Latvian. The following examples can be given: searching for information on travel restrictions in other countries and requirements for the crossing of borders (e.g., Poland and Sweden), using international sources which do not publish information in Latvian (e.g., the Health Organization, the Health and Safety Executive, which is the UK governmental agency), and communicating with international colleagues (e.g., Germany, Belgium, and Sweden). Most of these sources and contacts do not provide information and/or communicate in Russian, therefore, the data of the Central Statistical Boards were checked to identify which other languages might have been used. These data show that in 2017, 37.5% of the inhabitants in Latvia know the English language, 7.9%—German, and .9%—French (11). Based on these results, we assumed that the foreign language used for obtaining information on COVID-19 by employers and OSH experts was English. The above-mentioned data from the Central Statistical Bureau of Latvia allowed us to focus our article on the knowledge of the English language without looking at German, Spanish, French, or other most spoken languages in the world.

## Results

### Quantitative Results

The results of the surveys of OSH experts show that the percentage of respondents who have mentioned that they use only one language (Latvian) for searching professional information in OSH is between 25% (in 2006) and 35% (in 2018). Overall, the tendency shows that in 2006, every fourth respondent searched information in only one language, while it was every third respondent in 2018 (for details see Table 2). Looking at the data from the survey carried out in 2018 in more detail (the survey closest to the beginning of the COVID-19 pandemic), 35.3% of respondents have mentioned that he/she searched information in one language, 40.3% in two languages, and 23.9% in three languages (Latvian was one of those languages in both cases). In addition, 1 person has mentioned the use of four languages for a professional literature search.

**Table 2:** The number of languages mentioned by occupational safety and health (OSH) experts to specify the languages they use to search information in OSH.

**Number of languages**	**2006**		**2010**		**2018**	
	***n***	**%**	***n***	**%**	***n***	**%**
1 language	21	25.0	66	31.4	71	35.3
2 languages	28	33.3	86	41.0	81	40.3
3 languages	32	38.1	56	26.7	48	23.9
4 languages	2	2.4	2	1.0	1	0.5
5 languages	1	1.2	–	–	–	–
Number of respondents in total	84		210		201	

When trying to identify the respondents who searched information only in Latvian, we focused the analyses on the survey data of 2018. Such respondents were more often observed among the more experienced OSH experts (38.8% with experience between 6 and 10 years, and 40.2% with experience of more than 11 years). The lowest percentage of OSH experts reporting the use of only one language was observed among respondents working for the state authorities (the Ministry of Welfare, the State Labour Inspectorate) with 28.6%. Among OSH experts working in/with companies, the best situation was observed in the case of external OSH experts. Approximately, 31.9% of the respondents working in companies providing external OSH services mentioned Latvian as the only language to obtain information on OSH. Among external OSH experts, or service providers working in several companies, the relevant percentage was 35.7% (for additional information see Table 3).

**Table 3:** Characteristics of OSH experts using only Latvian for the search of OSH information by experience and workplace.

**Question**	**Grade**	**Only in Latvian**								
		**2006**			**2010**			**2018**		
		***n*** *	***N*** **	**%**	***n*** *	***N*** **	**%**	***n*** *	***N*** **	**%**
How long do you work	1–5 years	10	29	34.5	33	91	36.3	14	55	25.5
in OSH?	6–10 years	5	27	18.5	18	61	29.5	19	49	38.8
	More than 11 years	5	24	20.8	9	43	20.9	33	82	40.2
	At this moment I do not work in OSH	1	4	25.0	6	15	40.0	5	15	33.3
Where do you work?	In a company providing external OSH services	3	16	18.8	11	34	32.4	22	69	31.9
	In the OSH department of one company (there are more than one OSH expert in the company)	3	10	30.0	12	32	37.5	14	35	40.0
	In one company as an	4	17	23.5	14	47	29.8	13	34	38.2
	internal OSH expert (the only OSH expert in this company)									
	In several companies as an internal OSH expert	2	8	25.0	7	33	21.2	17	42	40.5
	In several companies as an external OSH expert (service provider)	1	4	25.0	5	16	31.3	5	14	35.7
	In state authority (Ministry of Welfare, State Labour Inspectorate)	6	20	30.0	6	16	37.5	2	7	28.6
	Other or unemployed	2	5	40.0	11	17	64.7	4	17	23.5

* *n—number of cases who have selected the particular answer out of respondents belonging to the group*.

** *N—number of respondents belonging to the group*.

In 2018 (the year of the survey closest to the beginning of the COVID-19 pandemic), English, as the foreign language used for searching of professional information, was mentioned by 42.8% of respondents (53.6% in 2006; 38.6% in 2010), while Russian was used by 46.8% respondents (58.3% in 2006; 54.8% in 2010). When looking at the experience of working in OSH, English was used most often by those specialists who hold a postgraduate degree in OSH but are not currently working in OSH (53.3%). From those who are working in OSH, the English language was the most frequently reported by those who have OSH experience of up to 5 years (50.9% in 1–5 years; 46.9% in 6–10 years). Respondents working as internal OSH experts (26.2%) and for the external OSH services (37.7%) less frequently reported the use of English (for details see Table 4). When analyzing the use of Russian, this answer was most often mentioned among persons whose experience is 1–5 years (50.9%) and more than 11 years (50%), those who work in/with several companies as internal OSH experts (57.1%), in a company providing OSH services (55.1%), and in several companies as an external OSH expert-service provider (50%) (for details see Table 5).

**Table 4:** Characteristics of OSH experts using English for the search of OSH information by experience and workplace.

**Question**	**Grade**	**English**								
		**2006**			**2010**			**2018**		
		***n*** *	***N*** **	**%**	***n*** *	***N*** **	**%**	***n*** *	***N*** **	**%**
How long do you work	1–5 years	17	29	58.6	28	91	30.8	28	55	50.9
in OSH?	6–10 years	17	27	63.0	27	61	44.3	23	49	46.9
	More than 11 years	9	24	37.5	20	43	46.5	27	82	32.9
	At this moment I do not work in OSH	2	4	50.0	6	15	40.0	8	15	53.3
Where do you work?	In a company providing external OSH services	11	16	68.8	19	34	55.9	26	69	37.7
	In the OSH department of one company (there are more than one OSH expert in the company)	5	10	50.0	10	32	31.3	17	35	48.6
	In one company as an internal OSH expert (the only OSH expert in this company)	9	17	52.9	15	47	31.9	16	34	47.1
	In several companies as an internal OSH expert	2	8	25.0	9	33	27.3	11	42	26.2
	In several companies as an external OSH expert (service provider)	3	4	75.0	7	16	43.8	7	14	50.0
	In state authority (Ministry of Welfare, State Labour Inspectorate)	9	20	45.0	7	16	43.8	3	7	42.9
	Other or unemployed	4	5	80.0	8	17	47.1	8	17	47.1

* *n, number of cases who have selected the particular answer out of respondents belonging to the group*.

** *N, number of respondents belonging to the group*.

**Table 5:** Characteristics of OSH experts using Russian for the search of OSH information by experience and workplace.

**Question**	**Grade**	**Russian**								
		**2006**			**2010**			**2018**		
		***n*** *	***N*** **	**%**	***n*** *	***N*** **	**%**	***n*** *	***N*** **	**%**
How long do you work	1–5 years	14	29	48.3	50	91	54.9	28	55	50.9
in OSH?	6–10 years	15	27	55.6	30	61	49.2	20	49	40.8
	More than 11 years	17	24	70.8	29	43	67.4	41	82	50.0
	At this moment I do not work in OSH	3	4	75.0	6	15	40.0	5	15	33.3
Where do you work?	In a company providing external OSH services	8	16	50.0	15	34	44.1	38	69	55.1
	In the OSH department of one company (there are more than one OSH expert in the company)	4	10	40.0	17	32	53.1	13	35	37.1
	In one company as an internal OSH expert (the only OSH expert in this company)	12	17	70.6	25	47	53.2	15	34	44.1
	In several companies as an internal OSH expert	5	8	62.5	24	33	72.7	24	42	57.1
	In several companies as an external OSH expert (service provider)	2	4	50.0	9	16	56.3	7	14	50.0
	In state authority (Ministry of Welfare, State Labour Inspectorate)	11	20	55.0	7	16	43.8	3	7	42.9
	Other or unemployed	4	5	80.0	12	17	70.6	7	17	41.2

* *n, number of cases who have selected the particular answer out of respondents belonging to the group*.

** *N, number of respondents belonging to the group*.

#### Qualitative Results on the Use of Foreign Languages for Searching Information

Direct or indirect use of English was mentioned by 21 employers (32.2% of all employers who participated in the focus group discussions) and 6 OSH experts (25%), resulting in a total of 27 focus group participants (30.3%). However, two employers and one OSH expert mentioned the use of foreign languages in general. Considering that both employers represent small companies from the region close to the border of Russia (the OSH expert did not provide any additional specific information), we were not able to specify which language was used in those three cases, therefore, we did not code these answers as an indirect use of English. None of the participants directly mentioned the use of Russian.

More often, the researchers were able to identify the use of foreign languages for obtaining information on COVID-19 indirectly than directly. The following examples can be used for describing direct and indirect identification of the use of foreign languages:

“*In English, we checked the information of … crossing the borders.”*

—A large company from Latgale

“*We are an international company, so our experience is based on information received from different countries—Sweden, Finland, China, Brazil, and each country had its examples.”*

—A small company from Riga, suburbs of Riga

In addition, the representatives from international companies, who explained that they received information and guidelines at the group level, have another indirect use of foreign languages as related to searching international experience to manage the COVID-19 pandemic or the trustful information from international organizations:

“*We started to look for international experience already before WHO [World Health Organization] guidelines. We analyzed in details experience from other countries, including China.”*

—A large company from Riga, suburbs of Riga

Another important reason for searching information in foreign languages was the need to obtain specific information from other countries:

“*As I was abroad during that time, I, of course, checked the website of the Ministry of Foreign Affairs and compared this information with Czech Republic, Poland, Germany.”*

—A small company from Vidzeme

#### Qualitative Results on Searching of Information

Five main themes of discussions were revealed: (1) looking for information in different channels, (2) looking for information from Latvian sources, (3) looking for information from international sources, (4) communication used for information search, and (5) problems with information (see Table 6).

**Table 6:** Themes identified during research analysis of focus group discussions (*n* = number of persons for which theme was detected).

**Main category**	**Subcategory**	**Employers**	**OSH experts**	**In total**
		**(***n*** = 65)**	**(***n*** = 24)**	**(***n*** = 89)**
Looking for information in different	Legal acts (online publications)	11	0	11
channels	Latvian news (evening news, online portals)	7	4	11
	Social media	6	2	8
	Press conferences of the government	4	0	4
	Foreign news	1	1	2
Looking for information from Latvian	Center for Disease Prevention and Control	24	12	36
sources	National working life portal stradavesels.lv	7	12	19
	State Labour Inspectorate	9	3	12
	Ministry of Health	3	4	7
	Center for Protection of Consumer Rights	3	1	4
	Ministry of Economy	2	2	4
	State Tax Authority	3	0	3
	Local authorities	3	0	3
	Ministry of Foreign Affairs	2	0	2
Looking for information from international	World Health Organization	6	6	12
sources	European Agency for Health and Safety at Work	0	2	2
	Other sources with a focus on experience from China	2	0	2
	Health and Safety Executive	0	1	1
Communication used for information	Latvian colleagues	5	8	13
search	International colleagues	8	2	10
	Industry-specific non-governmental organizations	7	3	10
	Internally hired experts (occupational physician, epidemiologist, a medical doctor specialized in infectious diseases)	2	1	3
	International clients	2	0	2
Problems with information	Low quality of information	8	1	9
	Too big amount of information	4	0	4
	Problems with reaching authority	2	2	4
Languages used for searching information	English	21	6	27
(other than Latvian)	Other languages than Latvian (in general)	2	1	3

In general, the sources used for obtaining information differed as to the needs of focus group participants differed. However, the general information on COVID-19, which was used by almost all participants, was mainly obtained from the news channels (TV, radio, online media, and links on social media):

“*It was enough with TV, radio and such internet news portals as* *www.delfi.lv*, *www.tvnet.lv*.”

—A small company from Latgale

“*Everything is on Facebook, Twitter, and they duplicate information 2, 3, 5 times.”*

—A large company from Vidzeme

However, other respondents mentioned that they used online publications of legal acts, as these sources contained more precise and reliable information. Participants also noted that watching live streams of the governmental press conferences was a very quick way to obtain information on changes in legal acts:

“*About COVID, we relied only on* *likumi.lv* *[the official publication site of legal acts in Latvia]. Information published in mass media was not precise, and this affects our business.”*

—A small company from Kurzeme/Zemgale

“*The necessary information was obtained through Facebook streaming of governmental press conferences.”*

—A small company from Latgale

For more business-oriented information, the participants used different options to obtain information from the national state authorities; in most cases, publications on their website. Although a designated website for COVID-19 related topics (12) was created, it was not among the most frequently used websites. As the top source, the Center for Disease Prevention and Control (CDPC) was mentioned by the employers. It was also one of the top two mentions by OSH experts:

“*We used a very simple tactic, and we followed it strictly. We acted as CDPC had said. If something was not clear, we wrote to CDPC and asked what to do. If the information is published on their website, we use it.”*

—A small company from Riga, suburbs of Riga

“*The first website for me was CDPC…”*

—An external OSH expert

The other top mention of OSH experts was the national working life portal (www.stradavesels.lv), which was already the main website for OSH topics even before the COVID-19 pandemic:

“*… I noticed and very much used the section of materials of the* *www.stradavesels.lv*. It was possible to find many informative materials in a single place on COVID.”

—An external OSH expert

Some other state authorities and local governments were specified less often; e.g., the State Labor Inspectorate, the Ministry of Health, the Center for Protection of Consumer Rights, the State Tax Authority, the Ministry of Economy, and the Ministry of Foreign Affairs. The number of the respondents specifying the particular institution was much lower and, in most cases, very specific information was searched in these cases. For example, the website of the Center for Protection of Consumer Rights was used to find information on personal protective equipment as this authority is responsible for the market surveillance of personal protective equipment, while the State Tax Authority was consulted for grants of current assets or stoppage benefits as this institution was coordinating these issues. For supporting quotes, see Table 7.

**Table 7:** The most relevant quotes to support the categories.

**Supporting quotes**	**Reference to the author of the quote**
**Languages used for searching information (other than Latvian)**	
“*The biggest input was from the group level. At the first the local situation was identified, then the guidelines come with information on what we need to focus on.”*	A large company from Kurzeme/Zemgale
“*Taking into account the fact that we have sites all over the world, almost in all continents, we exchanged with experience. We find out what they do, implement something similar, they take [ideas] from us. In OSH it is the same.”*	An internal OSH expert
“*We consulted with our [sister] company in Germany. They collaborated with a university hospital which summed up all up-to-date information and informed on the tendencies. That was the source we could trust.”*	A large company from Kurzeme/Zemgale
“*If we talk about the lack of information, then we did not wait for anything from the state*. … *We are an international company, so our experience is based on information received from different countries—Sweden, Finland, China, Brazil, and each country had its examples.”*	A small company from Riga, suburbs of Riga
“*We started to look for international experience already before WHO guidelines. We analyzed in details experience from other countries, including China.”*	A large company from Riga, suburbs of Riga
“*As I was abroad during that time, I, of course, checked the website of the Ministry of Foreign Affairs and compared this information with Czech Republic, Poland, Germany. What was the development of the situation there…”*	A small company from Vidzeme
“*We searched for information in the website of the Swedish authorities on what to do with the worker with transportation back to Latvia, what to do with him in Sweden [if he is COVID positive], can he live in the same house as other workers, etc.”*	A small company from Kurzeme/Zemgale
“*In English, we checked the information of … crossing the borders.”*	A large company from Latgale
**Other languages than Latvian (in general)**	
“*Yes, on the Internet. We looked for general information, what is going on in the other parts of the world.”*	An external OSH expert
“*Yes, in foreign languages we were checking the situation in Europe and … looking for creative solutions.”*	A large company from Latgale
“*The information in foreign languages was used either for private use or business interests, but everything is so similar to what you can find in Latvian.”*	A small company from Latgale
**Looking for information in different channels**
**Legal acts (online publications)**	
“*About COVID, we relied only on [the official publication of legal acts in] likumi.lv. Information published in mass media was not precise, and this affects our business.”*	A small company from Kurzeme/Zemgale
**Latvian news (evening news, online portals)**	
“*It was enough with TV, radio and such internet news portals as www.delfi.lv, www.tvnet.lv.”*	A small company from Latgale
“*Of course, that was press and internet. We adapted the available information from the original sources to our needs.”*	An external OSH expert
“*All sources of national mass media seemed to be very aggressive.”*	A small company from Vidzeme
**Social media**	
“*Everything is on Facebook, Twitter, and they duplicate information 2, 3, 5 times, and sometimes it is impossible to read all of it.”*	A large company from Vidzeme
“*Probably [the main source of information was] Facebook, I am logged in all the time.”*	A small company from Kurzeme/Zemgale
**Press conferences of the government**	
“*I want to add, that, I think that the communication of the government was rather clear. We followed online press conferences and used this information to draft our internal guidelines.”*	A small company from Vidzeme
“*The necessary information was obtained through Facebook streaming of governmental press conferences.”*	A small company from Latgale
**Foreign news**	
“*Information differed if it was obtained from CDPC, what is said by Dr. Apinis [former president of the Latvian Association of Physicians], what is said on CNN. These are different things.”*	An external OSH expert
**Looking for information from the Latvian sources**	
**Center for Disease Prevention and Control (CDPC)**	
“*The first website for me was CDPC…”*	An external OSH expert
“*CDPC… that was [the source of] information which is trustful… We can rely on this information.”*	An internal OSH expert
“*We used a very simple tactic, and we followed it strictly. We acted as CDPC had said. If something was not clear, we wrote to CDPC and asked what to do. If the information is published on their website, we use it.”*	A small company from Riga, suburbs of Riga
“*In Latvia, we used information from CDPC, we did not surf on the internet, different websites, and Facebook, as there is so much fake information around.”*	A large company from Kurzeme/Zemgale
**National working life portal (****www.stradavesels.lv**)	
“*There are informative materials in the [website] www.stradavesels.lv, a possibility to filter them and a special filter for COVID.”*	An external OSH expert
“*… I noticed and very much used the section of materials of the www.stradavesels.lv. It was possible to find many informative materials in a single place on COVID.”*	An external OSH expert
**State Labour Inspectorate**	
“*In March [of 2020] we used the website of the State Labour Inspectorate. Also, the telephone where they provide consultations.”*	A large company from Vidzeme
“*For OSH information and compulsory health surveillance, we checked the website of the State Labour Inspectorate. We have used this website on an everyday basis before COVID and continued to do the same during the pandemic.”*	A small company from Kurzeme/Zemgale
**Ministry of Health**	
“*We mainly used the written text of the website of the Ministry of Health and CDPC.”*	An external OSH expert
“*If the information is published on the website of CDPC, then we use it. The same with the website of the Ministry of Health.”*	A small company from Riga, suburbs of Riga
**Center for Protection of Consumer Rights**	
“*I have participated in some [online seminars] of the Center for Protection of Consumer Rights [on suitable personal protective equipment against COVID] and the Ministry of Economics.”*	An external OSH expert
**Ministry of Economy**	
“*We looked for information on the website of the Ministry of Economics…”*	A small company from Vidzeme
“*In the website of the Ministry of Economics, we looked for information on financial benefits for those companies who were on stoppage.”*	A small company from Latgale
**State Tax Authority**	
“*I think I obtained most information from the website of the State Tax Authority because the company was on stoppage. That was the only valuable source.”*	A small company from Vidzeme
**Local authorities**	
“*… then the local authority of Valmiera has issued some information…”*	A small company from Vidzeme
**Ministry of Foreign Affairs**	
“*They called the Ministry of Foreign Affairs and the Office of Citizenship and Migration Affairs as they employee foreigners from Ukraine and they needed information regarding their employment.”*	An external OSH expert
“*In principle, we followed information from CDPC and the Ministry of Foreign Affairs—mainly regarding restrictions of movement and traveling.”*	A small company from Vidzeme
“*As I was abroad during that time, I, of course, checked the website of the Ministry of Foreign Affairs and compared this information with Czech Republic, Poland, Germany. What was the development of the situation there…”*	A small company from Vidzeme
**Looking for information from the international sources**	
**World Health Organization**	
“*… And then there were also cases when we looked for something in English—Work Health Organization—in that website. There was more information on how to dress workers [in healthcare], what protection should be used.”*	An external OSH expert
“*The most precise information, of course, came from the World Health Organization. It was more related to health. I liked this source.”*	An external OSH expert
“*We obtained the basic information from the World Health Organization. Then some additional information came from CDPD. Both sources were trustful.”*	A small company from Riga, suburbs of Riga
**European Agency for Health and Safety at Work (EU-OSHA)**	
“*We followed the official sources, e.g., the website of the World Health Organization, EU-OSHA. Is there any new information available? Any new posters? Advice…”*	An external OSH expert
**Other sources with a focus on experience from China**	
“*We were looking for the experience of other countries, including China. Maybe it sounds silly, but we looked for measures that mitigate spreading of the virus, use, and placement of personal protective equipment, the flow of persons.”*	A large company from Riga, suburbs of Riga
**Health and Safety Executive (HSE)**	
“*[We used the same sources] as others—CDPD, the Ministry of Health, then websites in English, e.g., HSE.”*	A small company from Riga, suburbs of Riga
**Communication used for information search**	
**Latvian colleagues**	
“*Colleagues were the first source of information. Then scientific evidence.”*	An internal OSH expert
“*A very big support was our colleagues—mainly OSH experts. You communicate with one and then with another, so the ideas are generated.”*	An internal OSH expert
“*…and we also consulted colleagues from [the capital] Riga who work in the same area.”*	A small company from Vidzeme
**International colleagues**	
“*I have some clients—international companies. They had online cooperation with Germans and Scandinavians. They were very much engaged in topics related to COVID. When mother [company] implemented measures, we followed the same way.”*	An external OSH expert
**Industry-specific non-governmental organizations**	
“*… if we look at the professional websites, then each of the English versions starts with information on COVID—what measures, how to prevent, what is good, what is bad, In principle, these are the sources where we get the ideas.”*	An internal OSH expert
**Internally hired experts (occupational physician, epidemiologist, a medical doctor specialized in infectious diseases)**	
“*We have an occupational physician working in the external OSH service provider.”*	An external OSH expert
“*At the group level, experts, specialists of infectious diseases were engaged. They drafted guidelines and they were implemented by the regional departments.”*	A large company from Kurzeme/Zemgale
**International clients**	
“*Then we also had the information provided by our international clients. The clients sent their internal guidelines—what they do in their companies, and we also used that information.”*	A small company from Kurzeme/Zemgale
**Problems with information**	
**Low quality of information**	
“*Yes, there was so much information, so contradicting information.”*	An internal OSH expert
“*Unprecise information in mass media was republished.”*	A small company from Kurzeme/Zemgale
“*The information in Latvian Internet is very crippled.”*	A large company from Kurzeme/Zemgale
“*I have read very many publications. I was very much interested in the topic. I read information not only in Latvian but also Russian. So—information was available, but it was also so contradicting.”*	A small company from Vidzeme
“*The problem we faced was related to the fact that private media published information faster than official sources.”*	A small company from Latgale
**Too big amount of information**	
“*The problems were caused by the amount of information. In addition, there were many opinions, there were fewer answers to questions.”*	A small company from Latgale
“*Plenty of information… To verify the most essential one—it could be one of the problems.”*	A small company from Riga, suburbs of Riga
“*You must know how to use Internet resources, there are so many good things published there, but of course, …, you must also know how to filter it, which information is ok, which is not ok.”*	An external OSH expert
“*The amount of information was big, but you had to work hard to extract the specific information relevant to our industry. It had to be done yourself.”*	A small company from Vidzeme
**Problems with reaching authority**	
“*I had a reference from one company—it was impossible to reach them [the State Labour Inspectorate] by phone. Very long waiting time. That applied not only to the Labour Inspectorate, but also the Office of Citizenship and Migration Affairs.”*	An external OSH expert
“*In March, we searched the website of the State Labour Inspectorate and the Ministry of Welfare. Also, their consulting phone. It was really hot; I don't know who could reach them.”*	A large company from Vidzeme
“*I like original sources. Therefore, I still have the phone number of CDPD in my contacts. And if I had questions, I directly called them. Of course, sometimes it was difficult to reach them.”*	An external OSH expert

When analyzing the international sources of information, the World Health Organization was the most often mentioned source both by the employers and the OSH experts:

“*The most precise information, of course, came from the World Health Organization. It was more related to health. I liked this source.”*

—An external OSH expert

Other international sources included the European Agency for Health and Safety at Work (mentioned during the focus group discussion of external OSH experts) and the Health and Safety Executive, which is a UK governmental agency. In addition, several participants did not specify any particular source. However, they explained that they focused their search on the information describing the experience from China (supporting quotes are given in Table 7).

Along with searching information, communication with colleagues, clients, hired experts (an occupational physician, an epidemiologist, and a medical doctor specialized in infectious diseases), and industry-specific non-governmental organizations were mentioned. Both communications with persons in Latvia (with OSH experts or persons in the same industry) and abroad (colleagues from mother/sister companies and international clients) were reported:

“*A very big support was our colleagues—mainly OSH experts. You communicate with one and then with another, so the ideas are generated.”*

—An internal OSH expert

“*Taking into account the fact that we have sites all over the world, almost in all continents, we exchanged with experience. We find out what they do, implement something similar, they take [ideas] from us. In OSH it is the same.”*

—An internal OSH expert

“*Then we also had the information provided by our international clients. The clients sent their internal guidelines—what they do in their companies, and we also used that information.”*

—A small company from Kurzeme/Zemgale

Analysis of the transcripts of the focus group discussions allowed us to identify the main problems with obtainable information during the first wave of the COVID-19 pandemic in Latvia. The most typical answer was related to the quality of the information, though, it was mainly characterized as contradictory.

“*… information was available, but it was also so contradicting.”*

—A small company from Vidzeme

A big amount of information was mentioned by focus group participants, raising linked questions which were regarding the credible sources of information and the sufficient skills to distinguish the trustful sources. However, in general, these aspects were mentioned by a relatively small number of focus group participants and mainly by employers:

“*The problems were caused by the amount of information. In addition, there were many opinions, there were fewer answers to questions.”*

—A small company from Latgale

Several participants (both employers and OSH experts) also reported problems with reaching authority:

“*I had a reference from one company—it was impossible to reach them [the State Labour Inspectorate] by phone. Very long waiting time. That applied not only to the Labour Inspectorate, but also the Office of Citizenship and Migration Affairs.”*

—An external OSH expert

“*In March, we searched the website of the State Labour Inspectorate and the Ministry of Welfare. Also, their consulting phone. It was really hot; I don't know who could reach them.”*

—A large company from Vidzeme

## Discussion

The quantitative results of this study show that the percentage of OSH experts searching for professional OSH information only in the Latvian language is increasing. This is a very worrying tendency for the labor market in Latvia because the global working environment is rapidly changing. It has already been well recognized even before the COVID-19 pandemic that not only the OSH experts but the occupational health practitioners are also faced with constant changes in their working life (in companies or clinical practice, respectively) (13). If the general day-to-day job functions may not demand the use of good knowledge of the language, unusual circumstances may suddenly make the use of literacy skills critical (4). As an example of such unusual circumstances, a health or safety incident or emergency has been mentioned (14). According to our understanding, the COVID-19 pandemic can be described as a very unusual circumstance, which required the companies to take actions to mitigate the spread of the SARS-CoV-2 virus, and have shown how crucial OSH is for protecting the workers' health, for the functioning of our society, and the continuity of critical economic and social activities (15).

So, what is the danger of not being able to read and communicate in English? The possible answers can be identified from the results of the focus group discussions that the participants gave to describe if they used English for searching the information. The OSH experts, who are unable to read and understand English, to read trustful information published by international organizations, to obtain and analyze international experience, therefore, are less prepared to manage unusual circumstances. When these persons are unable to identify the needed information themselves, they must rely on other persons who can translate it, and, therefore, obtaining of information is delayed (time is needed for translation), or even changed/ modified (intentionally or unintentionally by the translator). Then, the next question is who should provide the translation. Also, the answer to this question can be found in the analyses of the focus group discussions, wherein the participants searched for information from the state authorities and colleagues. Although state authorities have strengthened their consulting capacity during the first wave of the COVID-19 pandemic in Latvia (16), there are still problems reaching them. This means that these persons were also in a disadvantaged position to get the answers to their questions. In addition, the drafting and approval of the documents to be published by the state authorities is usually a long and complicated process involving reaching an agreement between different involved parties. However, the time was critical in the case of COVID-19. The quality of the translation is also a question to be raised as it has been recognized in other areas (e.g., health care) that the lack of well-trained interpreters can adversely affect the health of individuals with low and limited English proficiency (17).

The focus group participants have mentioned informal communication with colleagues to obtain information related to COVID-19. This raises the question about the formal communication and involvement of two existing Latvian OSH-related non-governmental organizations. None of the focus group participants mentioned any of these organizations. We searched the websites of both organizations [one uniting companies—external OSH service providers (Association of Companies Providing External Occupational Safety and Health Services^1^) and one uniting individuals - OSH experts (Latvian Association of Occupational Health and Safety Specialists^2^)] and did not identify any of the published information related to COVID-19 since March 12, 2020, when the first emergency state was announced in Latvia.

When analyzing the workplaces of OSH experts, it initially seems that the best situation was observed in the case of external OSH experts; the lowest percentage of these experts reported obtaining professional OSH information only in Latvian. However, when looking closer to the data, a problem can be identified; Russian was mentioned more often than English (for companies proving OSH services: 55.1 vs. 37.7%; internal OSH experts in several companies: 57.1 vs. 26.2%) or equally as frequent for individual external OSH service provides (50%). Identifying that the Russian language is more used by OSH experts is a critical problem for Latvia, as the EU member state, because experts who are not using English were unable to search relevant information from EU and other European countries. The information on OSH is not published in Russian and, therefore, these specialists can only rely either on Latvian sources or communication with colleagues from Latvia. Obtaining relevant OSH information from other European countries is essential to get inspiring ideas for effective prevention of workplace hazards, thus, reducing the numbers of accidents at work and occupational diseases. The UK and its Health and Safety Executive can be mentioned as an example, as this afore-mentioned government agency and other OSH professional institutions published a huge amount of OSH-related information. They also reported one of the lowest fatal workplace accident rates (in 2018, the fatal workplace accident rate in the UK was .78% per 100,000 persons employed, while 3.27% per 100,000 persons employed in Latvia, with an EU average of 1.77% per 100,000 persons employed) (18). In addition, this group of external OSH experts is essential as the number of workplaces in companies that they can influence with their OSH consultation is big. According to the results of the *Working conditions and risk in Latvia* in 2018, about 50.6% of external OSH experts worked with up to 10 companies, 19.3% with 10–20 clients, 5.4% with 21–30 clients, 8.4% with 31–40 clients, and 16.3% with more than 40 clients (10).

The lack of highly qualified and knowledgeable OSH experts has also been recognized by companies providing external OSH services. According to the information published on the website of the non-governmental organizations uniting these companies, this organization has surveyed the opinion of their members. Among the main conclusions of this survey, a lack of qualified personnel is mentioned (19). Therefore, it is essential that the companies, the OSH service providers, strengthen their internal training systems. There is also room for non-governmental organizations to strengthen their capacities in providing training and formal communication for their members. In addition, these non-governmental organizations could provide more specialized information as the state authorities will never have enough capacity to provide very precise sector-specific information.

Looking at the relevant studies from other non-English speaking countries, English language knowledge has already been recognized as a vital competence in many companies and workers in the OSH context even before the COVID-19 pandemic. For example, the English language skills were identified in playing an essential role in the working environment and with OSH in Indonesia (20), but these conclusions have been based on other aspects. The study states that sufficient skills of the English language are “more required for communicating to expatriates, leading the meeting, doing the presentation, writing reports and designing Standard Operational Procedure.” A survey in the Philippines has shown that the low language proficiency of employees in the workplace limits their growth potential in the workplace and negatively impacts worker safety (21).

However, the knowledge of English among OSH experts has not been widely addressed. On one hand, when looking at different sources describing the competencies needed for OSH experts, the knowledge of languages is not covered. There can be several reasons for that; e.g., the Institution of Occupational Safety and Health, which has published “Competency framework. Professional standards for safety and health at work” (2), is a global organization for health and safety professionals based in the UK, where English knowledge for OSH experts is apparent. On the other hand, the English language knowledge should be an integral part of other competencies, e.g., knowledge management, which includes a continuous collection of information and facts relating to OSH (2). Such valuable information can originate not only from inside the company, where the OSH expert works but also outside of this company and even outside the country (2, 22). Thus, the OSH experts must be open-minded and ready for continuous learning and changing their work methods and networking in professional and business circles (23).

When discussing the possible steps to improve the English language knowledge, several methods should be suggested based on our findings. Firstly, additional efforts should be made to raise awareness of employers on the importance and added value to the OSH, and on the general business performance of the company in case the company employs well-educated OSH experts. Therefore, during the recruitment process of OSH experts, attention should be paid if the applicant has solid knowledge and skills of general OSH vocabulary (20). Secondly, it should be mentioned that one of the simplest and most efficient ways is to invest in the development of a specialized language training program by designing the English for Specific Purposes course for OSH experts (20). Good results can be achieved by creating a contextual curriculum and lesson plans, using a variety of innovative and attractive activities according to the interests of the OSH experts, and discussing topics that are close to their professional environment (24, 25). Thirdly, the general digital skills of OSH experts should be improved through training that includes the use of different machine translation services and language technologies which are becoming common in everyday practice for a variety of reasons and purposes (26).

When looking at the results of our study in terms of limitations, we have identified several of them. The main limitation of the web survey is the use of the non-probability sampling method to gather responses from OSH experts. Such a method had been used by legal entities in gathering data within the original studies, the *Work conditions, and risks in Latvia*. The surveys of OSH experts were carried out based on the public procurement process with requirements as predefined by the customer (the Ministry of Welfare in 2006, the Employers' Confederation of Latvia in 2010, and the State Labor Inspectorate in 2018). The authors of this article did not have any possibility to influence the survey methodology. In addition, the number of survey respondents is rather low (especially in 2006, with 86 respondents). At the same time, it is impossible to assess the percentage of OSH experts who have participated in the surveys as there is no national registry for OSH experts, which makes it impossible to calculate. The requirement to survey the opinion of 200 participants per survey in 2010 and 2018 has been specified by the client during the procurement process without any scientific calculations for sample size. In addition, several respondents in each survey mentioned that they search for information in English and Russian, which made the analyses more complicated. However, we concentrated our research on the advantages and disadvantages that came along with the use of both languages. Despite these limitations, the obtained results provide descriptive data and useful insights regarding the languages used by OSH experts in Latvia for searching professional information.

Another limitation is related to the time when the surveys and the focus groups were conducted. There is a time gap between 2018, which is the year of the newest survey data, and the onset of the COVID-19 pandemic in 2020, when the search for information was essential. Despite this time gap, it is clear that improving English language proficiency to a level that might be sufficient for searching reliable information on professional topics takes hundreds of hours (27); however, the OSH experts are adults with full-time jobs, family obligations, etc. In addition, we also added data from previous surveys (in 2006 and 2010) to find out the tendency and the speed of improvements. Nevertheless, we observed that the percentage of OSH experts searching for professional OSH information only in Latvian has increased. Therefore, we believe that the results obtained in 2018 provide indicative data to conclude that the percentage of OSH experts searching for professional OSH information in English is not sufficient and further actions should be taken.

There are also several limitations to focus group discussions. The first is related to the decisions of the Latvian government on legal requirements on mitigation of the spread of the SARS-CoV-2 virus. These requirements were used after the beginning of the first focus group discussion; therefore, some of the participants were onsite, while others were online. Although experienced moderators tried to provide equal opportunities for all participants (e.g., questions were asked to the focus participants individually), the equality of every single participant might have been affected.

Some problems were also faced while trying to recruit the focus group participants from companies that are not known as good practice examples for OSH management. Most of the focus group participants represented companies that can present good examples in most aspects of the health, safety, and wellbeing of their workers, who are also very keen to share their activities and experience. Therefore, the recruited participants might have overrepresented the companies with well-established OSH management systems, and the situation with the use of foreign languages among OSH experts could be even worse.

## Conclusions and Recommendations

Businesses have recognized health and safety benefits when literacy and/or language skills development is introduced in the workplace. Although this has been concluded about workers and not on OSH experts, we believe that knowledge of the English language for OSH experts working in small European countries is essential. Our study shows that knowledge of the English language for OSH experts working in Latvia is not sufficient to be ready for rapid changes in the working environment. Knowledge of the English language among OSH experts should be promoted through: (1) the recruitment process of OSH experts where attention should be paid if the applicant has solid knowledge and skills of general OSH vocabulary; (2) the development of a specialized language training program and designing the English for Specific Purposes course for OSH experts; and (3) improvement of digital skills of OSH experts with training that includes the use of different machine translation services and language technologies. Along with these processes, the companies providing external OSH services should establish a well functioning internal training system to provide their non-English speaking experts with up-to-date information. In addition, the OSH-related non-governmental institutions should strengthen their capacity to support state authorities in sharing information not only in an emergency like the COVID-19 pandemic but also related to other OSH aspects.

## Data Availability Statement

The data analyzed in this study was obtained from the owners of the original datasets of the independent *Work Conditions and Risks in Latvia* studies (the Ministry of Welfare, 2006, the Employers' Confederation of Latvia, 2010, and the State Labour Inspectorate, 2018), the following licenses/restrictions apply: requests to access these datasets must first be approved by the respective owners. Requests to access these datasets should be directed to the Ministry of Welfare (https://www.lm.gov.lv/en), the Employers' Confederation of Latvia (https://lddk.lv/en/), and the State Labour Inspectorate (https://www.vdi.gov.lv/en), respectively.

## Ethics Statement

The studies involving human participants were reviewed and approved by Ethics Commission of Riga Stradiņš University (Protocol No. 6-1/08/16, 23 July 2020). Written informed consent for participation was not required for this study in accordance with the national legislation and the institutional requirements.

## Author Contributions

IV and LM: conceptualization and methodology. LP and LA: software. LM, IV, and LP: formal analysis. LP and LM: investigation. LM: writing—original draft preparation and project administration. ME and IV: writing—review and editing. LA: visualization. IV: supervision. All authors have read and agreed to the published version of the manuscript.

## Funding

The focus group discussions and transcribing of the focus group discussions were funded by the National Research Programme of Latvia within the Project Life with COVID-19: Evaluation of Overcoming the Coronavirus Crisis in Latvia and Recommendations for Societal Resilience in the Future (Agreement No: VPP-COVID-2020/1-0013). The original datasets of the independent studies *Work conditions and risks in Latvia* were received from the data owners free of charge.

## Conflict of Interest

The authors declare that the research was conducted in the absence of any commercial or financial relationships that could be construed as a potential conflict of interest.

## Publisher's Note

All claims expressed in this article are solely those of the authors and do not necessarily represent those of their affiliated organizations, or those of the publisher, the editors and the reviewers. Any product that may be evaluated in this article, or claim that may be made by its manufacturer, is not guaranteed or endorsed by the publisher.

## Footnotes

^1^Association of Companies Providing External Occupational Safety and Health Services (in Latvian). https://dakib.lv/ (accessed November 30, 2021).

^2^Latvian Association of Occupational Health and Safety Specialists (in Latvian). https://5fc8de614e83d.site123.me/ (accessed November 30, 2021).
